# Computer Vision Tools for Low-Cost and Noninvasive Measurement of Autism-Related Behaviors in Infants

**DOI:** 10.1155/2014/935686

**Published:** 2014-06-22

**Authors:** Jordan Hashemi, Mariano Tepper, Thiago Vallin Spina, Amy Esler, Vassilios Morellas, Nikolaos Papanikolopoulos, Helen Egger, Geraldine Dawson, Guillermo Sapiro

**Affiliations:** ^1^Department of Electrical and Computer Engineering, Duke University, Durham, NC 27708, USA; ^2^Institute of Computing, University of Campinas, 13083 Campinas, SP, Brazil; ^3^Department of Pediatrics, University of Minnesota, Minneapolis, MN 55455, USA; ^4^Department of Computer Science and Engineering, University of Minnesota, Minneapolis, MN 55455, USA; ^5^Department of Psychiatry and Behavioral Sciences, Duke University, Durham, NC 22708, USA; ^6^Department of Psychiatry and Behavioral Sciences and School of Medicine, Duke University, Durham, NC 27708, USA; ^7^Department of Electrical and Computer Engineering, Department of Computer Science, and Department of Biomedical Engineering, Duke University, Durham, NC 27708, USA

## Abstract

The early detection of developmental disorders is key to child outcome, allowing interventions to be initiated which promote development and improve prognosis. Research on autism spectrum disorder (ASD) suggests that behavioral signs can be observed late in the first year of life. Many of these studies involve extensive frame-by-frame video observation and analysis of a child's natural behavior. Although nonintrusive, these methods are extremely time-intensive and require a high level of observer training; thus, they are burdensome for clinical and large population research purposes. This work is a first milestone in a long-term project on non-invasive early observation of children in order to aid in risk detection and research of neurodevelopmental disorders. We focus on providing low-cost computer vision tools to measure and identify ASD behavioral signs based on components of the Autism Observation Scale for Infants (AOSI). In particular, we develop algorithms to measure responses to general ASD risk assessment tasks and activities outlined by the AOSI which assess visual attention by tracking facial features. We show results, including comparisons with expert and nonexpert clinicians, which demonstrate that the proposed computer vision tools can capture critical behavioral observations and potentially augment the clinician's behavioral observations obtained from real in-clinic assessments.

## 1. Introduction

The analysis of children's natural behavior is of key importance for the early detection of developmental disorders such as autism spectrum disorder (ASD). For example, several studies have revealed behaviors indicative of ASD in early home videos of children that were later diagnosed with ASD [1–5]. These studies involved video recording infant behavior and then coding and analyzing the data a posteriori, using frame-by-frame viewing by an observer who typically trains for several weeks to achieve interrater reliability. Hours of labor are required, thereby making such analyses burdensome for clinical settings as well as for big data studies aiming at the discovery or improvement of behavioral markers. While clinical tools for early screening of ASD are available, they require administration and interpretation by specialists. Many families in low resource communities lack easy access to specialists in ASD. This work examines the potential benefits that computer vision can provide for research in early detection of ASD risk behaviors. It is a first milestone in a long-term project aimed at developing low-cost, automatic, and quantitative analysis tools that can be used by general practitioners in child development and in general environments to identify children at risk for ASD and other developmental disorders.

Although much is unknown about the underlying causes of ASD, some neuropathological studies indicate that ASD may have its origins in abnormal brain development early in prenatal life [6]. Moreover, Zwaigenbaum et al. [7] argue that many children with ASD exhibit several specific behavioral markers as early as in the first year of life. In high-risk siblings of children who later developed ASD, some of these symptoms can be observed during activities involving visual attention and are often expressed as difficulties in disengagement and shifting of attention [8, 9]. In addition, high-risk infant siblings have been shown to demonstrate impaired visual attention development between the ages of 7 and 14 months [10]. With this said, there is still much research needed to be done in determining potential risk indices. For example, a study performed by Nadig et al. [11] found that there is no significant difference in response-to-name disengagement between at-risk and low-risk infants.

Despite the fact that autism symptoms often emerge early and the syndrome can be diagnosed in toddlers, the average age of ASD diagnosis in the USA is close to 5 years [12]. Recently, research has demonstrated the benefit of early detection and diagnosis to allow for early intensive intervention. Early intervention, initiated in preschool and sustained for at least 2 years, can substantially improve child outcomes [13]. Detecting ASD risk and starting interventions before the full set of behavioral symptoms appears to may ultimately have an even greater impact, preventing difficult behaviors and delayed developmental trajectories from taking hold [14]. Although the diagnosis of ASD involves much more than the detection of symptoms, improving availability of cost-effective and accessible methods for identifying which children might be at risk and in need of further evaluation would potentially be of value. Towards this end, we have focused on developing semiautomatic computer vision video analysis techniques to aid in measuring ASD-related behaviors which can be used in early detection research.

More specifically, the main objectives in this paper are to use and validate computer vision tools to capture reliably two critical visual attention behaviors, Disengagement of Attention and Visual Tracking, belonging to the Autism Observation Scale for Infants (AOSI) [15], a behavioral observation tool for gathering information on early ASD risk signs [16]. (In this paper we refer to the AOSI and the scoring based on the DSM-IV. Research is needed to assess how measures of early signs of autism derived from automated video coding, such as those from the AOSI, relate to later diagnosis of ASD based on the newly established DSM-5 criteria. This is the subject of on-going efforts in our team.) Thus, the aim of the study is to examine the correspondence between the measures derived from the computer vision tools and the clinical assessment given by one trained expert. To demonstrate the validity of our tools, we compare our computer vision methods' results to those of the AOSI trained expert who performed the assessments and three nonexperts. Towards this end, other objectives of our work are to demonstrate the accurate and objective measurements provided by our low-cost methods and their potential to be used in the research of ASD risk marker identification. The work with such a specific population of infants and toddlers is unique in the computer vision community, making this a novel application for the psychology community. While the data is obtained from actual clinical assessments, the tasks pulled from the assessment are easy to administer and/or involve recordings of the child's natural behavior, thereby opening the door to broad behavioral studies, considering that the actual analysis is automatically done as introduced here.

These tools could potentially aid the practitioner and researcher in the risk marker identification task by providing accurate and objective measurements. These measurements can further provide means for improving the shareability of clinical records without compromising anonymity. In addition and particularly for research, automatic analysis will permit researchers to analyze vast amounts of naturally recorded videos, opening the door for data mining towards the improvement of current assessment protocols and the discovery of new behavioral features. This project is being developed by a multidisciplinary group bringing together professionals from psychology, computer vision, and machine learning. As an alternative to other research strategies [17–19], which require laboratory assessments, one of our main goals is to provide nonintrusive capturing systems that do not necessarily induce behavioral modification in the children. In other words, hardware must not constrain the testing environment; for example, the children are not asked to wear any type of sensors [20, 21].

## 2. Methods

### 2.1. Procedures for AOSI Tasks

The AOSI consists of a set of tabulated tasks that are designed for assessing specific behaviors, where each task consists of a certain number of presses and the child's responses receive scores. According to the AOSI, Disengagement of Attention is characterized as the “ability to disengage and move eyes/attention from one of two competing visual stimuli” [15, 22], while Visual Tracking is characterized as the “ability to visually follow a moving object laterally across the midline” [15, 22]. During the AOSI assessment, the clinician performs three trials for the Disengagement of Attention task and two trials for the Visual Tracking task, per participant. Every trial receives an AOSI-tabulated score, according to the following guidelines. 

*Disengagement of Attention*. This activity consists of (1) shaking a noisy toy to one side of the infant until his/her attention is engaged and then (2) shaking a second noisy toy on the opposite side, while continuing to shake the first object. A delayed response in high-risk infants has been shown to be associated with a later ASD diagnosis [7, 15]. A trial is considered “passed” if the child looks to the second object in less than 1s, considered “delayed” if the child looks after a 1-2 s delay, and considered “stuck” if the child looks after more than 2 s.

*Visual Tracking*. To evaluate this activity, the following is performed: (1) a rattle or other noisy toy is used to engage the infant's attention, (2) the rattle is positioned to one side of the infant, and (3) the rattle is then moved silently at eye level across the midline to the other side. In high-risk infants, an interrupted, delayed, or partial gaze tracking has been shown to be associated with a later ASD diagnosis [15]. Depending on how continuously and smoothly the participant is able to track the object, the trial is considered “passed,” “delayed or interrupted,” or “partial or no tracking.”

The clinician makes a “live” judgment about these time frames or may look at videos of this task if available. Finally, an overall score for each task is computed by merging the individual trials. We followed the protocol of comparing the assessments done by (1) an expert psychologist examiner who has been trained in the AOSI as well as ASD diagnosis in children, (2) a child/adolescent's psychiatrist, (3) two psychology students with no particular autism training, and (4) the results of our new computational tools. The child/adolescent's psychiatrist and two psychology students assigned their scores by following the AOSI guidelines, without prior training, while watching the same videos used by the automatic method. This setup allows us to contrast the automatic method's findings with human assessments across the full range of expertise.

### 2.2. Computer Vision Algorithms for Assessing Visual Attention

**Figure 1:**
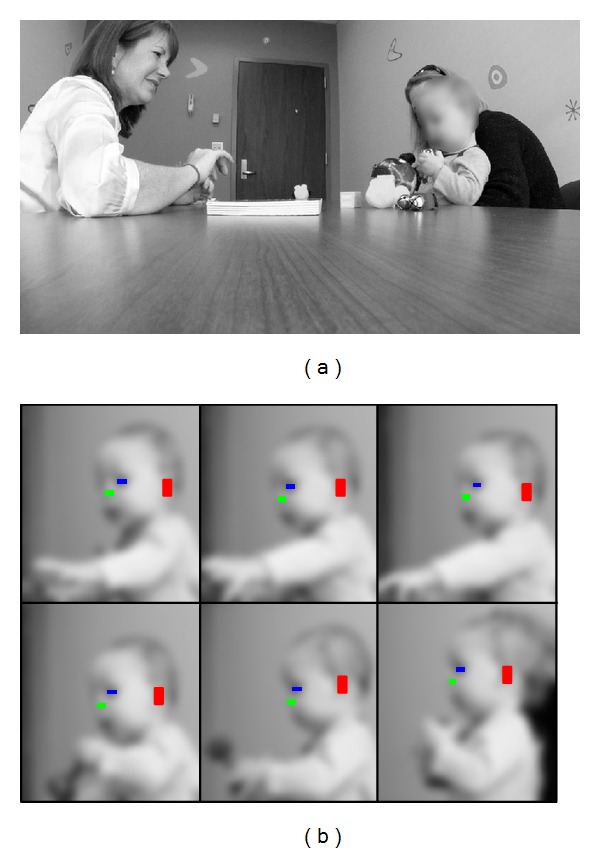
(a) General scene capturing the AOSI evaluation session. (b) Example of our algorithm automatically tracking three facial features: the left eye, left ear, and nose. In this paper, all figures have been blurred to protect the participants' privacy.

To analyze the child's reactions in the Visual Attention activities, we automatically estimate the changes of two head pose motions: yaw (left and right motion) and pitch (up and down motion). For the Visual Tracking and Disengagement of Attention tasks, which involve lateral motions, we focus on the yaw motion. We develop computer vision algorithms for estimating these head motions from low-cost cameras. The algorithms track specific facial features: the left ear, left eye, and nose (see right image of Figure 1). From their positions we estimate the participant's yaw and pitch motions. The only user input in our algorithm is during initialization. On the first frame, the user places a bounding box around the left ear, left eye, and nose (Figure 7). This could potentially be avoided by standard feature detection techniques. We marked the play objects by hand, although this also can be done automatically from prior knowledge of their visual and sound features (e.g., color or squeaking noise). Additional technical details are available in Appendices A and B (Figure 6 presents the data flow of our visual attention analysis system). We should note that we exploit and extend computer vision techniques that form the basis of virtual all automatic face analysis systems, and therefore have been extensively validated in the literature.

Scoring for our automatic method is based on automatic visual inspection of the estimated head motion measurements. After marking when the second object is presented in the Disengagement of Attention task, our method is able to automatically determine when the participants start and complete disengagement from the first object to the second. We assign the disengagement delay based on how many frames/seconds it takes the participant's head motion to completely transition to the second object (note that we are recording the video at 30 frames per second). We incorporate a + (1/3) of a second margin for each delay to accommodate human error of making a live judgment. The scoring for the Visual Tracking task is determined by visual inspection of the head motion measurements as the object is moving laterally in front of the participants. More specifically, the scores are assigned based on whether or not the measurements exhibit full lateral head motion and also depend on the rate of change of the measurements. A “pass” is assigned if the head motion measurements exhibit full lateral head motion and a smooth rate of change. If there is an instance where the measurements exhibit a plateau or the rate of change changes direction for a short period of time but the measurements still display full lateral head motion, an “interrupted” score is assigned. For trials where the measurements do not exhibit full lateral head motion, a “partial” or “no tracking” score is assigned. Examples of our method's measurements for a “pass,” “interrupted,” and “partial” or “no tracking” tracking scores are shown in Figure 3. The developed automatic technique operates at a much higher resolution and accuracy than the standard 1-second intervals used by the expert clinician during live testing.

### 2.3. Participants

The purpose of the study was not to examine the correspondence between early assessments and outcome but rather the ability of our tools to accurately capture individual differences in behavior. We sought to include a sample in which a diversity of responses to the AOSI would be expected; thus the sampled population of this study involves 12 at-risk participants being examined in a clinic, including both males and females ranging in age from 5 to 18 months. Approval for this study was obtained from the Institutional Review Board at the University of Minnesota, and we have gathered our data from a series of ASD evaluation sessions of an ongoing concurrent study performed on a group of at-risk infants, at the Department of Pediatrics of the University of Minnesota.

All at-risk participants were infant siblings of a child diagnosed with ASD, a premature infant, or as a participant showing developmental delays.  Table 1 presents a summary of this information. Note that, the participants are not clinically diagnosed until they are 36 months of age and only participant number 3 has presented conclusive signs of ASD.

### 2.4. Hardware

In our clinical setup, we use a low-cost GoPro Hero HD color camera (with a resolution of 1080 p at 30 fps), placed freely by the clinician in the center of the table between 2 and 4 feet away from the participant to ensure that it remains still throughout each trial and captures both the clinician and the participant (e.g., left image of Figure 1). The displayed images here are downsampled, blurred, and/or partially blocked to preserve anonymity (processing was done on the original videos).

## 3. Results

### 3.1. Disengagement of Attention

Table 2 summarizes the results of our method, the clinical assessments, and the ratings by a child/adolescent's psychiatrist and two psychology students for the Disengagement of Attention task. (See all video results in supplementary video files available online at http://dx.doi.org/10.1155/2014/935686.) Since the current set up for the visual attention tasks only involves a single camera placed nonintrusively, there are trials that our current method cannot handle (this could be easily solved in the future with a second low-cost camera). These trials include instances when the participant left the camera's field of view or when a toy or object obstructed it. (Standard face detection algorithms, such as the ones used in digital cameras, can be used to automatically alert the clinician of such cases for repositioning of the camera if needed.) For Table 2, the trials with blank spaces and a horizontal line correspond to such cases. Out of the 24 trials that the clinician assigned a “pass” score, our method agreed on 23 of them and scored a “delayed” for the other trial. And out of the 3 trials the clinician scored “delayed” our method agreed on 2 trials, scoring one as a “pass.” The clinician did not assign a score for number 2, stating that it was a “spoiled” trial due to the participant being afraid of the toys. However, we show our method's results to exemplify a possible Disengagement of Attention score.

**Figure 2:**
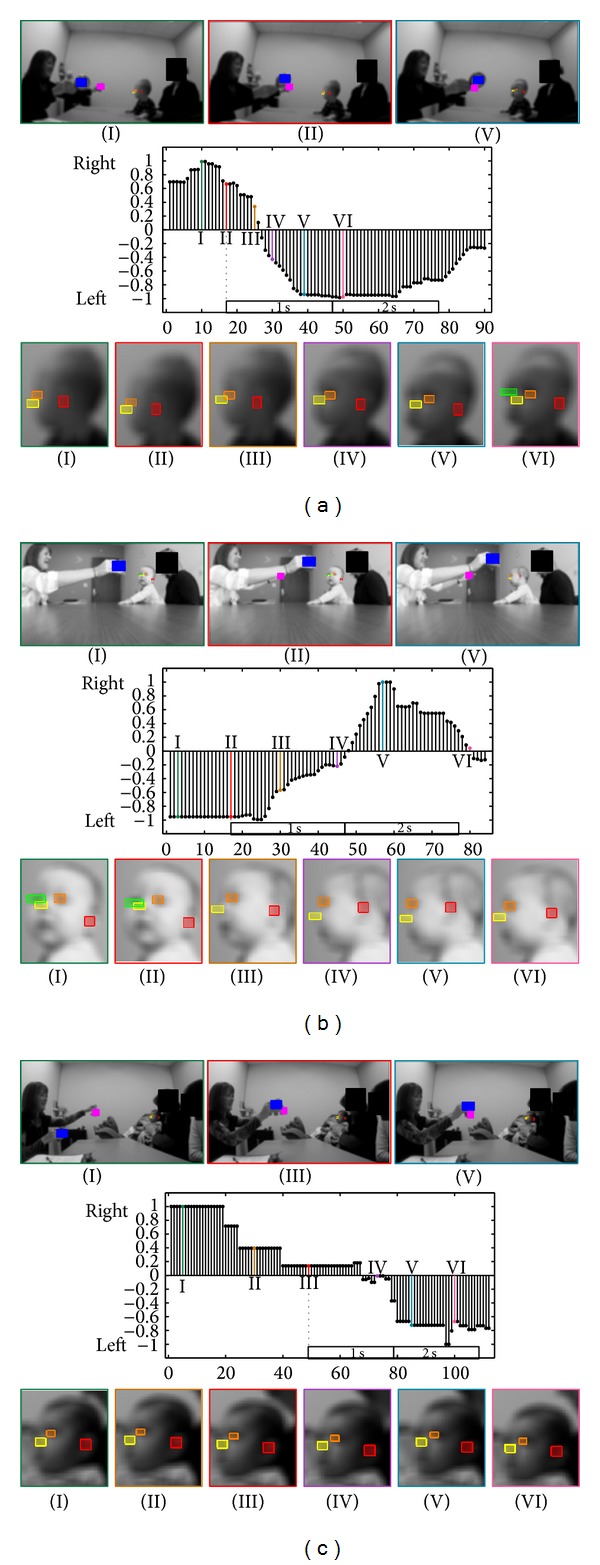
(a) First example of the Disengagement of Attention task. Top: when the clinician is holding one object, when the second object appears, and when the child recognizes the second object. Middle: changes in the yaw motion ($\hat{\text{yaw}}$ values in the *y*-axis; see Appendices A and B) for every frame (*x*-axis). The dotted line represents when the second object is presented, followed by boxes representing 1 and 2 seconds after the object is presented. Bottom: 6 examples of the infant's face during the task. All facial features are automatically detected and tracked (as indicated by the colored boxes around the nose, eyes, and ear). Colors and roman numerals identify corresponding images and spikes in the graph. (b, c) Two examples of the Disengagement of Attention task.

To further clarify our results, Figure 2 displays examples of our method's results and important cases for the Disengagement of Attention task. In Figure 2(a), the participant is able to disengage from the first object and look at the second within 0.7 s (21 frames) of the second object being presented. This would be scored as “passed” on the AOSI test. The participant in Figure 2(b) disengages to the second object within 1.3 s (40 frames), which would be scored as “delayed” on the AOSI test.

### 3.2. Visual Tracking

Table 3 summarizes the results of our method, the clinical assessments, and the ratings by a child/adolescent's psychiatrist and two psychology students for the Visual Tracking task. As in Table 2, the trials with blank spaces and a horizontal line could not be used by our automatic method. Out of the 14 trials that the clinician assessed as “pass”, our method agreed with 13 of them and scored an “interrupted” for 1 of the trials. For all the 4 trials the clinician assessed as “interrupted,” our automatic method was in agreement. The clinician scored two trials as “partial,” our method scored one of them as “partial” and the other as “interrupted.”

**Figure 3:**
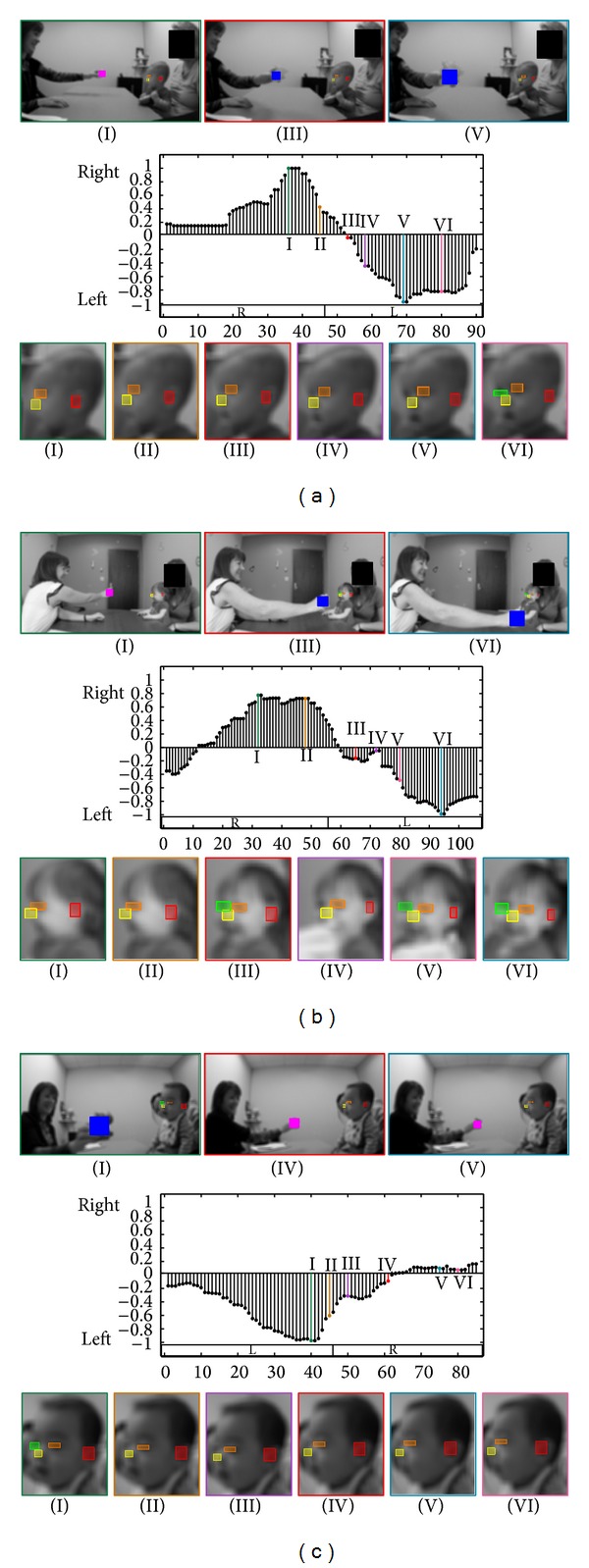
(a) First example of the Visual Tracking task. Top: when the clinician is holding the object, when the object is at one extreme side (right or left), and when the object is at the other extreme side. Middle: changes in the yaw motion ($\hat{\text{yaw}}$ values in the *y*-axis; see Appendices A and B) for every frame (*x*-axis). The boxes labeled “R” and “L” represent when the object is to the right and left of the participant, respectively. The gray shaded areas represent when the object is not moving and at an extreme side (either right or left). Bottom: 6 examples of the infant's face during the task. Colors and roman numerals identify corresponding images and spikes in the graph. (b, c) Two examples of the Visual Tracking task.

Figure 3 shows important examples of our results for the Visual Tracking task.  Figure 3(a) demonstrates a participant that received a “passed” on the AOSI's Visual Tracking task, since the participant was able to smoothly track the object with minimal delay as the object approached the participant's right. In Figure 3(b), the participant exhibited “interrupted” tracking motion. The participant's tracking of the object was interrupted as the object moved across the clinician's face. Instead of tracking the object as it moved across the clinician's face, the participant stopped tracking the object and looked at the clinician for 0.46 s (14 frames) before continuing to track the object as it moved to the participant's left. Such short behaviors can be detected by an automatic system. In Figure 3(c), the participant displays a “partial” tracking score on the AOSI. As the object crosses the clinician's face, the participant completely stops tracking the object and instead looks straight at the clinician.

### 3.3. Comparisons between Ratings by Automatic Computer Vision Method, Nonexpert Clinical Raters, and Expert Clinician

We next compared ratings made by nonexpert clinical raters (child/adolescent's psychiatrist and two psychology students) and by the computer vision methods with ratings made by an expert clinician. The results obtained by the child/adolescent's psychiatrist and two psychology students are presented in Tables 2 and 3. Out of the 27 Visual Disengagement trials (Table 2), the two psychology students agreed with the clinician on 13 and 16 of the trials, respectively, while the child/adolescent's psychiatrist agreed on 22 trials. The computer vision system agreed with the expert clinician in 25 out of the 27 cases. Similarly for the 22 Visual Tracking trials (Table 3), the two psychology students agreed with the expert clinician on 13 and 14 of the trials, respectively, while the child/adolescent's psychiatrist agreed on 16 trials. The computer vision system agreed on 19 of the 22 cases.  Table 4 shows the interrater reliability value for each individual compared with the expert clinician, based on weighted Cohen's kappa with a range of 0-1, where 1 means complete agreement.

## 4. Discussion

In addition to providing a broadly deployable low-cost tool for ASD risk assessment, if validated in future research, the potential benefits of an automated method for head motion estimation are threefold. First, it would provide accurate quantitative measurements for tasks assessing infant visual attention, such as the AOSI tasks, improving the shareability of clinical records while not compromising anonymity. Second, it could also prove beneficial in the discovery of new behavioral patterns by easily collecting large amounts of data and mining it. Third, it could increase the granularity of the analysis by providing data at a finer scale. As the results demonstrate, the computer vision method performed very well when compared to the expert clinician and outperformed the three other nonexperts. Using unweighted kappa, Bryson et al. [15] reported a combined average interrater reliability score of 0.80 for both the Disengagement of Attention and Visual Tracking. Although our combined average weighted score of 0.75 is not as high as reported by the original authors, it is still considered excellent (greater than 0.65) according to them.

### 4.1. Disengagement of Attention

Compared to the expert clinician's results, the computer vision method achieved high accuracy. In Table 2, the method obtained one false positive by scoring one trial “delayed” that the clinician scored as “pass” (participant number 6) and missed one “delayed” trial (participant number 11). In the current project, the temporal resolution is 30 frames per second, allowing for discovery of possible latent head motion patterns.  Figure 2(c) provides an interesting pattern in the participant's head movement. Not only does it take the third participant over 1 s to look at the second object (which is “delayed” on the AOSI), but the participant displays piece-wise constant lateral head movements compared to the other two examples (which presented a much smoother motion), a pattern virtually impossible to detect with the naked eye. Again, such automatic and quantitative measurements could potentially add critical information that could aid in risk detection, such as new ways of scoring the AOSI. With a study on a larger population, new time intervals (and their variability) for scoring may be discovered, and these false positives could be analyzed not as a strict “pass” or “delayed” but as something in between.

### 4.2. Visual Tracking

Again, compared to the expert clinician's results, the automated method achieved high accuracy. As shown in Table 3, the clinician scored one trial belonging to the only participant that has been positively diagnosed as “delayed”; however, based on our nonintrusive camera placement (as selected by the practitioner), it was not possible to continuously extract the object's location accurately enough to assign “delayed” scores. In future studies, it would be possible to extend the setup to include an overhead camera to accurately record the playing objects' positions. Another aspect of the method is that it provides accurate and quantitative measurements of the participant's head motions; thus, one is able to automatically determine the delays between when the participant looks at the object or how long the participant stops his/her tracking.

## 5. Concluding Remarks

This work is the first milestone in a long-term project focused on the development of noninvasive early observation of children in order to aid in risk detection and research of neurodevelopmental disorders. With the goal of aiding and augmenting the visual analysis capabilities in evaluation and developmental monitoring of ASD, we proposed (semi-) automatic computer vision tools to observe specific important behaviors related to ASD elicited during AOSI, providing both new challenges and opportunities in video analysis. The proposed tools, if validated in future research, could significantly reduce the effort by only requiring interactive initialization in a single frame. The eventual goal is to minimize the need for extensive training and add both accuracy of quantitative measurements and objectivity. We focused on two visual attention activities performed during the AOSI. We developed specific computer vision algorithms for these activities, obtaining encouraging results that correlated with an expert's judgment. The automated method proved to be more consistent with the expert clinician's rating that those produced by less expert human observers; it should be noted that these human observers still have higher levels of expertise than normally available in most school settings.

The improvement and extension of the proposed methods is an ongoing work and we plan to provide code for the method. Current limitations of our methods include requiring the participant's face to be present throughout the video, estimating the yaw and pitch motions independently of one another, and we have only tested on relatively high-definition video sequences (from low-cost GoPro Hero cameras). Although the present study included 12 at-risk infants and toddlers, some limitations of our study include the use of only a single expert examiner as well as the small and relatively unimpaired sample size (although as mentioned before, the exploited tools have been extensively validated in the literature). For the visual attention tasks, we plan to complement the estimation of the child's motions with estimating the red examiner's behaviors. The work presented demonstrates the validity of the tools with a specific examiner's assessments. To expand these tools to broader clinical and naturalistic settings that involve different examiners and examiners with different levels of training, the quality of interaction, engagement, and variability between the examiners must also be considered. These examiner-related behaviors include how far away from the participant the examiner positions the toy(s), the displacement velocity of the toy(s) throughout the assessment, audio cues from the examiner, and head position of the examiner in the sense of whether he/she is looking at the participant or away. A study on the variability of examiner's performance in both clinical and naturalistic settings, such as at the participant's home, is an important topic for future research and should be considered before any data mining analyses are carried out. A method that examines the examiner's behaviors would also allow the examiner to receive immediate feedback on whether a trial or press needs to be redone. Notice that this could also lead to an automatic method for training examiners. Based on the set-up of the AOSI tasks discussed in this work, we assume that the participant's head motion is directly correlated to his/her gaze direction. This assumption is known as “center bias” and has been well established in gaze estimation and saliency detection literature [23, 24]. To further research and broaden the scope of this method, we plan to validate the assumption of the direct relationship between the head motion and eye movements in a specific population of infants and children at risk for ASD or with ASD; see also [25] for some early results in this direction.

### 5.1. Extensions of Computer Vision Tools for ASD Behavioral Observations

**Figure 4:**
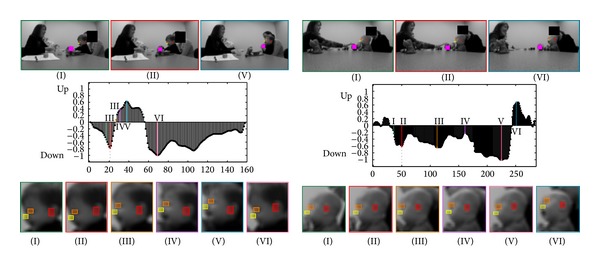
Two examples of the ball playing activity. Top: when the ball contacts the child, when the child looks down at the ball, and when the child looks up at the clinician. Middle: changes in the pitch motion (*y*-axis) for each frame (*x*-axis). The dotted line represents when the ball contacts the participant. Bottom: 6 examples of the infant's face during the administration. All facial features are automatically detected and tracked. Colors and roman numerals identify corresponding images and spikes in the graph.

There are additional potential behavioral risk indices for ASD, both included in and beyond the scope of AOSI, such as facial expression in first birthday home videos [26] and mounting a camera near the examiner's face to estimate the participant's gaze [27], which are not addressed by the current method but we aim to address in the future, both in terms of the technical methods and the assessment of their validity and reliability. Computer vision tools hold promise as a novel and cost-efficient method for capturing behavior that could be used in both the clinical and research settings. Using the methods in this work, we also provide initial data suggesting that these methods might be useful in less structured situations, such as tracking a participant's head motion during a ball playing activity (Figure 4), providing information regarding the participant's interaction with the examiner such as the time it takes for a participant to look up after receiving a ball.

**Figure 5:**
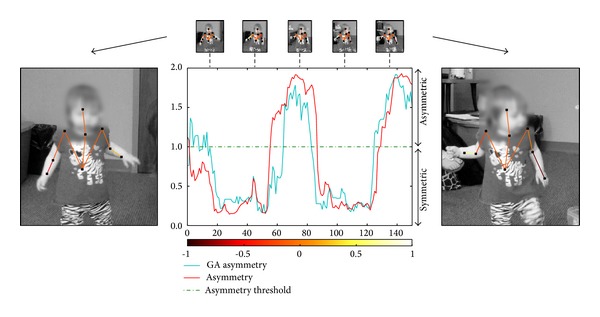
Comparison of arm-asymmetry scores between automatic method and ground truth in a video segment containing participant number 1. The cyan line represents normalized results from our method, while the red line represents the ground truth (GT) of the normalized arm differences. See [28] for more information on arm-asymmetry calculations and analysis. The normalized color scale visually displays the angle difference between the right and left forearms, where symmetric arm positions have similar overlaying colors.

**Figure 6:**
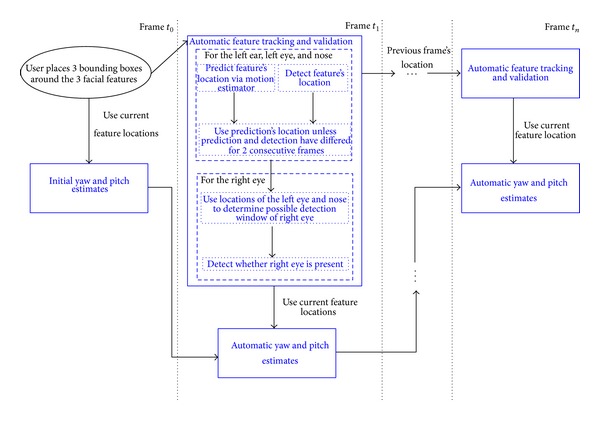
Data flow of the system for estimating yaw and pitch motions. We only require the user to place a bounding box around the left ear, left eye, and nose in the first frame of the video segment (black ellipse at time *t*_0_). All the subsequent steps occur in a fully automatic fashion (blue blocks).

**Figure 7:**
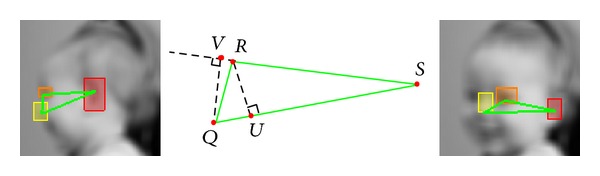
The triangle created by the left ear, left eye, and nose. The leftmost and rightmost images depict the triangle when the infant is looking right and more towards the camera, respectively. The middle image shows the points used for calculating $\hat{\text{yaw}}$.

In addition, computer vision tools are not restricted to only aiding visual attention related markers. Our group has also been developing nonintrusive tools for aiding the assessment of motor patterns [28]. Through a meticulous process of hand fitting stickman to the participants in every frame, Esposito et al. [29] have found that toddlers with ASD often presented asymmetric arm positions in early life. Using computer vision, we were able to automatically estimate the 2D body pose of the toddlers in the video segments (Figure 5) as well as estimate arm angles relative to the ground with little user intervention (see mentioned reference for technical details and evaluations). The only required user intervention consisted of interactively creating a segmentation mask in the initial frame of the video segment. By creating nonintrusive and semiautomatic computer vision tools, such as these, large datasets can be analyzed, potentially leading to the discovery of new and/or improved behavioral markers.

## Supplementary Material

The Supplementary Material provided has 3 videos containing our automatic method's results from the: Disengagement of Attention task, Visual Tracking task, and Ball Rolling Activity. The results of these videos are also in Tables 2 and 3 of the paper. Note that the videos are blurred and downsampled for the participants' privacy. The videos are in mp4 format (codec h.264) and have been tested using windows media player, quicktime, and VLC player.
The colors of the playing object(s) in the clinician hand(s) represent if the object is to the left or right of the participant. The colors of the bounding box around each participant's head, as well as the text above the bounding box, represent if the participant is looking to the left or right for the Disengagement of Attention and Visual Tracking tasks of the clinician according to our method. For the Ball Rolling Activity, the bounding box and colored text represent whether the participant is looking up or down according to our method.

## Figures and Tables

**Table 1:** Information on participants involved in this study. Each participant was chosen for a different reason: being a baby sibling of someone with ASD, a premature infant, or showing developmental delays.

Part number	Age (months)	Gender	Risk degree
Number 1	14	F	Showing delays
Number 2	5	F	Baby sibling
Number 3	16	M	Showing delays
Number 4	15	M	Showing delays
Number 5	8	M	Premature infant
Number 6	9	F	Premature infant
Number 7	10	F	Premature infant
Number 8	9	M	Premature infant
Number 9	7	M	Premature infant
Number 10	6	M	Baby sibling
Number 11	9	M	Premature infant
Number 12	18	M	Showing delays

**Table 2:** Results of Disengagement of Attention task. Scores from the clinician (Clin.), automatic method (Automatic), a psychiatrist (Psy.), and two students (St. 1 and St. 2) for each trial. A trial is considered either as “passed” (Pass), “delayed” (Del), or “stuck” (Stck) depending on whether the child disengages from the first object in less than 1 s, between 1 and 2 s, or more than 2 s, respectively. We also present the automatically computed delay that the child takes to disengage. Note that we consider *a* + (1/3) of a second margin for each delay to accommodate human error of making a live judgment.

Part number	Clin.	Automatic		Psy.	St. 1	St. 2
		Score	Delay (s)			
	First trial score					
Number 1	Pass	Pass	0.9	Pass	Del	Del
Number 2	N/A	Del	1.87	N/A	N/A	N/A
Number 3	Pass	Pass	0.5	Pass	Del	Del
Number 4	Pass	Pass	0.23	Pass	Pass	Pass
Number 5	Del	Pass	1.07	Del	Del	Del
Number 6	Pass	Pass	1.03	Pass	Del	Pass
Number 7	Pass	Pass	0.83	Pass	Del	Del
Number 8	—	—	—	—	—	—
Number 9	—	—	—	—	—	—
Number 10	Pass	Pass	0.87	Del	Del	Del
Number 11	Pass	Pass	0.83	Pass	Del	Pass
Number 12	Pass	Pass	0.93	Pass	Pass	Pass

	Second trial score					
Number 1	Pass	Pass	0.7	Pass	Pass	Pass
Number 2	—	—	—	—	—	—
Number 3	—	—	—	—	—	—
Number 4	Pass	Pass	1.1	Pass	Pass	Pass
Number 5	Del	Del	1.77	Del	Stck	Del
Number 6	Pass	Del	1.43	Pass	Pass	Pass
Number 7	Pass	Pass	0.97	Del	Del	Del
Number 8	Pass	Pass	1.33	Pass	Del	Del
Number 9	—	—	—	—	—	—
Number 10	Pass	Pass	1.3	Pass	Pass	Pass
Number 11	Pass	Pass	0.63	Pass	Pass	Pass
Number 12	Pass	Pass	0.9	Pass	Del	Del

	Third trial score					
Number 1	Pass	Pass	0.37	Pass	Pass	Pass
Number 2	—	—	—	—	—	—
Number 3	—	—	—	—	—	—
Number 4	Pass	Pass	0.3	Pass	Pass	Pass
Number 5	Pass	Pass	0.5	Pass	Del	Pass
Number 6	Pass	Pass	0.7	Del	Del	Del
Number 7	Pass	Pass	1.13	Pass	Del	Del
Number 8	—	—	—	—	—	—
Number 9	Del	Del	1.37	Pass	Del	Stck
Number 10	Pass	Pass	1.33	Del	Del	Del
Number 11	Pass	Pass	0.87	Pass	Pass	Pass
Number 12	Pass	Pass	0.87	Pass	Pass	Pass

**Table 3:** Results of Visual Tracking task. Scores from the clinician (Clin.), automatic method (Automatic), a psychiatrist (Psy.), and two students (St. 1 and St. 2) for each trial. A trial is considered “passed” (Pass), “delayed” (Del), “interrupted” (Int), “partial” (Prt), or “no tracking” depending on how smoothly the child visually tracks the object.

Part number	First trial score					Second trial score				
	Clin.	Automatic	Psy.	St. 1	St. 2	Clin.	Automatic	Psy.	St. 1	St. 2
Number 1	Pass	Pass	Pass	Pass	Pass	Pass	Int	Int	Int	Int
Number 2	Int	Int	Pass	Int	Pass	Int	Int	Pass	Pass	Pass
Number 3	Del	Pass	Pass	Pass	Pass	Pass	Pass	Pass	Pass	Pass
Number 4	Pass	Pass	Pass	Pass	Pass	Pass	Pass	Pass	Pass	Pass
Number 5	Prt	Int	Pass	Int	Pass	Prt	Prt	Del	Prt	Prt
Number 6	Pass	Pass	Pass	Pass	Pass	—	—	—	—	—
Number 7	—	—	—	—	—	Int	Int	Int	Prt	Prt
Number 8	Pass	Pass	Pass	Del	Pass	Pass	Pass	Pass	Pass	Pass
Number 9	Pass	Pass	Pass	Del	Pass	Pass	Pass	Pass	Pass	Pass
Number 10	Pass	Pass	Pass	Pass	Pass	Int	Int	Int	Prt	Prt
Number 11	Pass	Pass	Pass	Pass	Pass	Int	Int	Int	Prt	Prt
Number 12	Pass	Pass	Pass	Pass	Pass	Pass	Pass	Pass	Pass	Pass

**Table 4:** Number of agreements with the autism expert for each participant in the two visual attention tasks and overall interrater reliability using weighted Cohen's kappa. See Tables 2 and 3.

Task	Trials	Automatic	Psychiatrist	Student 1	Student 2
Disengagement	27	**25**	22	13	16
tracking	22	**19**	16	13	14

Total	49	**44**	38	26	30
Interrater score	—	**0.75**	0.37	0.27	0.27

## Appendices

### A. Tracking and Validating Facial Features

This section provides technical details about the algorithm for tracking facial features and computing head motions from them. The large variability of the data and the lack of control about the camera positioning call for using very simple and robust features and algorithms.

We assume that, in the first frame, we have bounding boxes of three facial features: the left ear, left eye, and nose (see, e.g., Figure 2). These bounding boxes are in practice selected by hand on the first frame. It is possible to achieve a fully automatic initialization, but this was not the objective of the present work. The user intervention is nonetheless minimal.

We aim at tracking these three facial features. Following a scheme loosely based on the TLD tracker [30], we use dense motion estimation coupled with a validation step that employs an offline-trained facial feature detector. The dense motion estimator [31] tracks the features with high accuracy in most cases, but when the child's head moves quickly, illumination changes can sometimes cause the tracker to lag behind the features. Thus, we validate the output of the tracker using facial feature detectors in every frame (Figure 6).

To validate the features we train left eye, right eye, left ear, and nose detectors. For this, we adapt the widely used method by Dalal and Triggs [32], proposed for pedestrian detection, to our particular setting (see also [33, 34]). Our method employs the popular multiscale Histograms of Orientated Gradients (HOG) using 8 × 8 pixel blocks and 9 orientation bins as descriptors to represent each facial feature and then classifies these descriptors using a support vector machine (http://www.csie.ntu.edu.tw/~cjlin/libsvm/) with a radial basis function kernel (see [32] for further technical details). As positive training samples, we use hand labeled facial patches from children in our experimental environment. As negative training samples, we extract random patches from around multiple children's faces. Our classifier was trained a single time before any experiment was carried out. Then, we used it for all experiments with no need for retraining or parameter setting.

For each frame, search areas for the facial feature detectors are defined around the bounding boxes given by the tracker. The left eye, left ear, and nose are present in every frame for the given camera position and their final detected positions are determined by the locations that exhibit a maximal response from the classifier (i.e., extrema of each feature's classifier output). The tracker's bounding boxes are validated if their centers are within the bounding boxes returned by the detectors; however, if the tracker's centers are outside of the detector's bounding boxes for two consecutive frames, then the corresponding bounding box for the tracker is reset to a new location within the detector's bounding box. Determining the presence of the right eye aids in the estimation of the yaw motion. The rectangular search area for the right eye, which is not tracked since it appears and disappears constantly due to the camera position, is based on the location of the detected nose and the horizontal and vertical distances between the detected left eye and nose. More specifically, the search area is between the detected nose's location plus/minus the horizontal and vertical distances between the detected left eye and nose. Also employed as a right eye search area restriction is that the nose must be between the left and right eyes.

Thus, using our method, we are able to track the facial features via a dense motion estimator, and validate their positions via the facial feature detectors. To estimate head motion, see the following, we use the facial feature locations given by the dense motion estimators. The dense motion estimator provides smoother and more precise locations than the detector.

### B. Yaw and Pitch Motion Estimation from Facial Features

As a way to provide an accurate motion estimation of the pitch angle, we cumulatively sum the vertical coordinate changes of the left eye and nose with respect to the left ear every frame. We expect a positive sum when the child is looking up and a negative sum when the child is looking down, with the magnitude representing how much the child is looking up or down.

For estimating the yaw motion, we calculate two ratios based on the triangle created by the left ear, left eye, and nose (Figure 7); we also use information about the presence of the right eye. Let *Q*, *R*, and *S* denote the locations of the nose, left eye, and left ear, respectively. For the first ratio *r*_NoseToEye_, we project *R* into the line defined by *QS*, thus defining the point *U*; we then define *r*_NoseToEye_ = |*US* | /|*QS*|, where |·| is the Euclidian distance. For the second ratio, we project *Q* into the line defined by *RS*, defining *r*_EyeToEar_ = |*VR* | /|*RS*|.

The two ratios *r*_EyeToEar_ and *r*_NoseToEye_ are inversely proportional. Looking at Figure 7, we can observe that when the face is looking in profile view, *r*_EyeToEar_ will be large and *r*_NoseToEye_ will be small, conversely when the face is in frontal view (looking more towards the camera). To combine these two ratios into one value, we calculate the normalized difference between them, $\hat{\text{yaw}}=(r_{\text{EyeToEar}}-r_{\text{NoseToEye}})/\left(r_{\text{EyeToEar}}+r_{\text{NoseToEye}}\right)$. Thus, as the child is looking to his/her left, $\hat{\text{yaw}}$ goes to −1; and, as the child is looking to his/her right, $\hat{\text{yaw}}$ goes to 1. The presence of the right eye further verifies that the infant is looking left.

We incorporate whether the right eye is present or not to verify that the infant is looking left or right at the maximum and minimum $\hat{\text{yaw}}$ values.
